# Evaluation of a national clinical programme for the management of self-harm in hospital emergency departments: impact on patient outcomes and the provision of care

**DOI:** 10.1186/s12888-023-05340-4

**Published:** 2023-12-07

**Authors:** G Cully, P Corcoran, D Gunnell, SS Chang, B McElroy, S O’Connell, E Arensman, IJ Perry, E Griffin

**Affiliations:** 1 School of Public Health, University College Cork, Cork, Ireland; 2 National Suicide Research Foundation, Cork, Ireland; 3 NIHR Biomedical Research Centre, University Hospitals Bristol and Weston NHS Foundation Trust, Bristol, UK; 4 Population Health Sciences, University of Bristol, Bristol, UK; 5 Institute of Health Behaviors and Community Sciences, and Global Health Program, College of Public Health, National Taiwan University, Taipei City, Taiwan; 6 Department of Economics, Cork University Business School, University College Cork, Cork, Ireland; 7 School of Applied Psychology, Australian Institute for Suicide Research and Prevention, Griffith University, Brisbane, QLD Australia

**Keywords:** Self-harm, Emergency department, Emergency psychiatry, Repetition, Assessment, Hospital services, Interrupted time series

## Abstract

**Background:**

Emergency departments are important points of intervention, to reduce the risk of further self-harm and suicide. A national programme to standardise the management of people presenting to the emergency department with self-harm and suicidal ideation (NCPSHI) was introduced in Ireland in 2014. The aim of this study was to evaluate the impact of the NCPSHI on patient outcomes and provision of care.

**Methods:**

Data on self-harm presentations were obtained from the National Self-Harm Registry Ireland from 2012 to 2017. The impacts of the NCPSHI on study outcomes (3-month self-harm repetition, biopsychosocial assessment provision, admission, post-discharge referral, and self-discharge) were examined at an individual and aggregate (hospital) level, using a before and after study design and interrupted time series analyses, respectively. The 15 hospitals that implemented the programme by January 2015 (of a total of 24 between 2015 and 2017) were included in the analyses.

**Results:**

There were 31,970 self-harm presentations during the study period. In hospitals with no service for self-harm (n = 4), risk of patients not being assessed reduced from 31.8 to 24.7% following the introduction of the NCPSHI. Mental health referral in this hospital group increased from 42.2 to 59.0% and medical admission decreased from 27.5 to 24.3%. Signs of a reduction in self-harm repetition were observed for this hospital group, from 35.1 to 30.4% among individuals with a history of self-harm, but statistical evidence was weak. In hospitals with a pre-existing liaison psychiatry service (n = 7), risk of self-discharge was lower post-NCPSHI (17.8% vs. 14.8%). In hospitals with liaison nurse(s) pre-NCPSHI (n = 4), medical admission reduced (27.5% vs. 24.3%) and there was an increase in self-harm repetition (from 5.2 to 7.8%. for those without a self-harm history).

**Conclusion:**

The NCPSHI was associated with improvements in the provision of care across hospital groups, particularly those with no prior service for self-harm, highlighting the need to consider pre-existing context in implementation planning. Our evaluation emphasises the need for proper resourcing to support the implementation of clinical guidelines on the provision of care for people presenting to hospital with self-harm.

**Supplementary Information:**

The online version contains supplementary material available at 10.1186/s12888-023-05340-4.

## Background

Previous self-harm is the strongest risk factor for suicide [1]. Among those who attend hospital following self-harm, 16% with will re-attend with a further act of self-harm within 12 months and 1.6% will die by suicide within five years [2]. As such, emergency departments are important points of intervention, to reduce the risk of further self-harm and suicide and to provide appropriate care and follow-up. The rate of hospital-presenting self-harm is approximately 200 per 100,000 [3, 4]. Such presentations are often complex and treatment needs can be physical as well as psychosocial [5]. It is well established that, despite there being existing quality care standards and guidance in the management of such presentations, there is significant variation in the treatment and management of self-harm in hospital emergency departments [6, 7]. This variation is largely explained by hospital, as opposed to clinical, factors – reflecting the availability of mental health resources and hospital policies [8]. Standardising the management of self-harm in hospital settings is critical in providing evidence-informed support and may reduce risk of both non-fatal and fatal repetition.

The National Clinical Programme for Self-Harm and Suicide-related Ideation (NCPSHI) in Ireland was introduced between 2014 and 2017, in an attempt to standardise the care and management of self-harm in general hospital settings [9]. The programme involved the training and integration of 35 specialist mental health staff (clinical nurse specialists) in emergency departments, whose role would be to provide a standardised and tailored care for adults presenting to hospital with self-harm. The NCPSHI model of care outlines a pathway of care for all adults, aged 18 years and over, who presented to the emergency department following self-harm or with suicidal ideation. This includes: (1) receiving an empathic, timely response in the emergency department; (2) receiving a biopsychosocial assessment; (3) ensuring family members are involved at the assessment and discharge planning and; (4) providing linkage to next appropriate care [9].

To our knowledge, there are no existing studies which examine the impact of a national programme such as the NCPSHI on hospital-presenting self-harm, with few looking at changes in service reconfiguration at a regional or hospital level [10–12, 39]. The current study is the first from a larger mixed methods study exploring both the impact and implementation of the NCPSHI between 2014 and 2017 in Ireland [13]. Using data from the National Self-Harm Registry Ireland [3], the aim of the current study was to evaluate the impact of the introduction of the NCPSHI on patient outcomes and provision of care.

## Methods

### Setting

There are 26 acute general hospitals in Ireland, providing a 24-h emergency department service, which were eligible to implement the NCPSHI. Within the Irish healthcare system, hospitals fall under seven geographical hospital groups, each with their own governance structure. The 26 acute hospitals are located across all seven hospital groups.

#### Intervention

As part of the NCPSHI (previously titled the National Clinical Programme for the Assessment and Management of Patients Presenting to the Emergency Department following Self-harm (NCP-SH)), a model of care [14] and standard operating procedures [15] were developed to standardise the clinical management of self-harm in emergency departments. This model of care was delivered through the placement of dedicated Clinical Nurse Specialists (CNSs) across eligible hospitals. It is the responsibility of the CNSs to implement the four components of the model of care. The NCPSHI was implemented in Ireland between 2014 and 2017 across a total of 24 hospitals. Initially, 15 hospitals implemented the programme between June 2014 and January 2015. The implementation of the programme across the remaining nine hospitals was more staggered, beginning between September 2015 and June 2017.

### Study design

The study is a natural experiment, using routinely collected data on self-harm presentations to the emergency department to examine the impact of the NCPSHI on patient outcomes, including changes in repetition of self-harm and changes in the provision of care in the hospital. This study uses two approaches to examine the impact of the NCPSHI on these outcomes: before and after study design and interrupted time series analyses. This study is reported in line with the Strengthening the Reporting of Observational Studies in Epidemiology (STROBE) guidelines [16]. A full description of the design has been described in a protocol for the wider study that this study is situated within [13].

A number of changes were made from the original study protocol [13]. Due to the staggered nature of the implementation of the NCPSHI, this analysis focuses on the 15 hospitals that initially implemented the programme, rather than all 24 hospitals, as indicated in the protocol. A start date of January 2015 was selected as all 15 hospitals had implemented the programme by this date. The implementation dates of the remaining hospitals ranged from September 2015 onwards with large gaps between each additional hospital. The inclusion of the selected hospitals was based solely on the date of implementation. Differences in the characteristics of the hospitals did not factor into the decision to include or exclude hospitals. Following publication of the protocol, data were accessed that indicated variation across hospital sites in the level of implementation of the NCPSHI, which was related to the services that were in place for assessing individuals who presented to the emergency with self-harm prior to the introduction of the programme. Therefore, the 15 included hospitals were classified into three groups according to the pre-existing services for assessing self-harm within the hospitals. Group 1 (n = 7) consists of hospitals which had a pre-existing designated liaison psychiatry service; Group 2 (n = 4) consists of hospitals which had a pre-existing liaison nurse(s) in; and Group 3 (n = 4) consists of hospitals which had no service for assessing self-harm. All analyses were stratified according to these groups. An overview of the characteristics of the hospitals and the NCPSHI implementation across the three groups is presented in Table 1. Given that self-harm history is an established strong predictor of repetition [2, 4], and has been shown to impact care received within the emergency department [17], we further stratified the analyses on self-harm repetition according to whether an individual had presented with self-harm in the previous 12 months or not. This outcome was also examined for the full sample, in line with the protocol, with findings reported in the supplementary material (Supplementary Tables 1 and 2). Finally, an additional outcome measure was added to the original analyses plan. The outcome of self-discharge was not included in the original protocol but was added due to its relevance as a potential indicator of the impact of the NCPSHI on the experience of self-harm patients in the emergency department.

### Data source

Data on hospital-presenting self-harm for adults aged 18 years and over were obtained from the National Self-Harm Registry Ireland (Registry), a national monitoring system of all attendances to hospital emergency departments in Ireland as a result of self-harm. The standard operating procedures of the Registry have been described previously [4, 18]. The definition of self-harm used by the Registry is ‘an act with non-fatal outcome in which an individual deliberately initiates a non-habitual behaviour, that without intervention from others will cause self-harm, or deliberately ingests a substance in excess of the prescribed or generally recognised therapeutic dosage, and which is aimed at realising changes that the person desires via the actual or expected physical consequences’ [19].

Data relating to the implementation of the NCPSHI in each hospital was gathered from multiple sources. The date of implementation at each hospital site, the number of CNSs appointed, details of any pre-existing service and the hours of service cover was determined through programme documentation, including site reports developed as part of an interim review of the operation of the programme, confirmed through discussions with the management team of the NCPSHI and correspondence with clinical staff in the participating hospitals [20]. Additional information on the pre-existing services within the hospitals was gleaned from a government report [21].

**Table 1:** Overview of hospital groupings, including hospital characteristics and details of NCPSHI implementation within each group

	Group 1 - Liaison psychiatry service (7 hospitals)	Group 2 - Liaison nurse (4 hospitals)	Group 3 - No service (4 hospitals)
**Hospital information**			
Pre-existing service / staff for self-harm	Designated liaison psychiatry service	Liaison nurse(s)	No designated staff
Hospital type^a^	Tertiary	General	General
City location	6 (90%)	0	0
Self-harm attendances per year, mean	530	236	241
**Details of implementation of NCPSHI**			
Date of implementation​	Jun - Dec 2014	Aug - Dec 2014	Nov 2014 - Jan 2015
Number of new staff appointments (FTE^b^), average n per hospital	1.2	0.80	1.5
NCPSHI cover, hours per day/days per week^c^	12+/7	8–9/5	12+/7
Out of hours cover in place	7 (100%)	1(25%)	4(100%)

a. Reflects hospital type for the majority of hospitals in given group. Hospital types are: general (hospitals that provide 24/7 acute medicine, surgery and critical care) and tertiary (hospitals that provide tertiary care in addition to the services of general hospitals) (42).

b. FTE = full time equivalent.

c. Reflects hours of cover for the majority of hospitals in given group.

### Outcome measures

The primary outcome measure was the proportion of self-harm presentations followed by a repeat presentation to any hospital emergency department nationally within 3 months (91 days), i.e. self-harm repetition, examined separately for those with and without a history of self-harm in the preceding 12 months. The main secondary outcome measure was the proportion of patients not receiving a biopsychosocial assessment. Non-assessment was examined due to the high proportions of assessments conducted across hospitals (average = 71%). The following secondary outcome measures relating to processes of care were also examined: the proportion of presentations resulting in admission to a medical or psychiatric inpatient ward; and the proportion of patients receiving a mental health referral post-discharge; the proportion of patients who self-discharged without being triaged or before a next care recommendation could be made.

### Covariates

Covariates obtained from the Registry comprised of sociodemographic and clinical variables. Sociodemographic variables included gender, age, and medical card status (whether the individual had access to free medical services, based on income and/or health status). Clinical variables included include method(s) of self-harm, alcohol involvement, time of attendance, arrival by ambulance or other emergency services, self-harm history and clinical management (including receiving a biopsychosocial assessment, medical and psychiatric admission, mental health referral). No information was available on the level of suicidal intent associated with self-harm presentations as this is not routinely collected as part of the Registry data.

### Statistical analysis

We conducted analyses at individual and aggregate level. At individual level, Poisson regression models were used to examine differences in the primary and secondary outcome measures before and after the implementation of the NCPSHI. We also used Poisson regression with interrupted time series analysis to test if the introduction of the NCPSHI impacted on the outcome measures at hospital level. Incidence rate ratios (IRRs) and their 95% confidence intervals (CIs) are reported for all models. We identified January 2015 as the start date of the NCPSHI in the 15 hospitals. We used at least a two-year pre and post period of observation for both individual and hospital-level models. For repetition, the analysis covered the time period January 2012 to December 2017. Due to data availability, the analysis for the secondary outcome measures were limited to January 2013 to December 2017.

For the individual Poisson regression models, all repeat presentations were included in the analyses, with each repeat presentation becoming an index presentation. Lack of independence of observations between presentations made by the same individual was adjusted for by using robust analyses that modified the variance of estimates. Adjusted analyses included sex and any covariates significantly associated with the outcome variable in univariable analyses, using a significance level of p < 0.2.

For the time series models we used a bi-monthly unit of analyses, resulting in 36 time points in the self-harm repetition models and 30 time points in the models examining the secondary outcome measures. We adjusted for seasonality by including a categorical variable representing bimonthly intervals. We assessed for autocorrelation by running population averaged models using a generalised estimating equation approach. We also adjusted for changes in rates of self-harm presentations over time. No evidence of autocorrelation was observed so the most parsimonious models are reported in the results. We also carried out sensitivity analyses to test whether there were differences when we excluded three time points (July to December 2014) and four time points (July 2014 to February 2015) to account for bedding in of the intervention and the findings were consistent. Rates of self-harm repetition and non-assessment were plotted for all relevant subgroups, and trend lines for the pre- and post-intervention period were estimated using the following coefficients from the time series regression models: base risk, pre-NCPSHI trend, risk change, and post-NCPSHI trend.

For all outcome measures, we conducted analyses separately according to the three hospital groups. Analyses using the full sample were also conducted and are included in supplementary Tables 1 and 2. We conducted additional time series models to test for interactions for the two main outcomes (self-harm repetition and non-assessment), which confirmed that the effect of the NCPSHI differed across the three hospital groups. For self-harm repetition, we further stratified the analyses by recent self-harm history, resulting in six groups for this outcome. Due to the increased probability of false positives associated with multiple testing, reported *p* values should be interpreted with caution. All analyses were conducted using SPSS IC V16.0 and Stata SE V17.

## Results

### Cohort characteristics

Between January 2012 and December 2017, there were 31,970 presentations to the emergency departments of the 15 study hospitals, involving 18,224 individuals. Half the presentations were made by females (52.2%), and the median age was 33 years (interquartile range 21 years) (Supplementary Table 3). The most common method of self-harm was intentional drug overdose (IDO), involved in two thirds of presentations (63.9%), followed by self-cutting (22.3%). Alcohol was involved in 36.5% of presentations. Of the 21,339 (66.8%) presentations involving individuals without a recent history of self-harm, 7.2% were followed by a repeat presentation within 3 months. Repetition was more common (33.5%) following presentations involving individuals with a recent history of self-harm. Over the full study period, risk of non-assessment was 28.9%, medical admission occurred following 23.2% of presentations and 10.2% resulted in psychiatric admission. Of those discharged from the emergency department, 44.8% received a mental health referral. Individuals self-discharged before receiving a referral in 15.4% of presentations.

In the three years before and after the implementation of the NCPSHI, there were a similar numbers of self-harm presentations made to the study hospitals (16,140 vs. 15,830). This was consistent across the hospital groups with similar numbers of presentations in the pre- and post-intervention period for Group 1 *Liaison psychiatry service* (10,956 vs. 10,798), Group 2 *Liaison nurse* (2,235 vs. 2,183), and Group 3 *No service* (2,949 vs. 2,849). Some differences were observed in the characteristics of self-harm presentations from the pre- to the post-intervention period; there was a decrease in the involvement of alcohol in presentations across all hospital groups and in presentations arriving to hospital by ambulance in groups 1 and 3, and an increase in presentations involving individuals with a medical card (Supplementary Table 4).

### Self-harm repetition

**Fig. 1:**
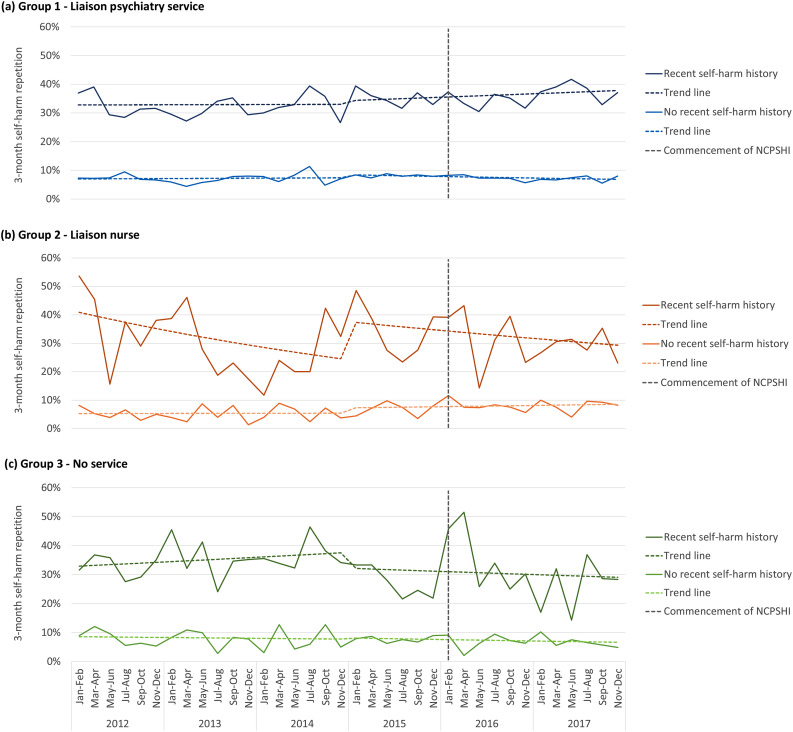
Self-harm repetition by hospital group among persons with and without a recent self-harm history

There were indications of a reduction in 3-month self-harm repetition following the introduction of the NCPSHI for Group 3 *No service* (Tables 2 and 3; Fig. 1). This was particularly apparent for those with a history of recent self-harm repetition in this hospital group, with a 14–15% reduction in repetition risk in the post-intervention period in both the individual level (adjusted incidence rate ratio (IRR) 0.85; 95% confidence interval (CI) 0.67–1.09) and hospital level analyses (0.86; 0.62–1.17). This pattern of reduced repetition for group is further illustrated in Fig. 1. There was evidence of an increase in 3-month self-harm repetition for hospital Group 2 *Liaison nurse*, particularly for those without a history of recent self-harm with an increase in repetition observed in the individual analysis for this group (1.48; 1.13–1.94) (Table 2). This pattern was observed to a lesser extent in the hospital-level analysis (1.35; 0.74–2.45) (Table 3; Fig. 1).

**Table 2:** Poisson regression models for self-harm repetition in the post- versus pre-NCPSHI period, by hospital group

	Self-harm repetition			
	**Pre-NCPSHI** n (%)	**Post-NCPSHI** n (%)	**Unadjusted IRR (**95% CI)	**Adjusted IRR** ^**a**^ (95% CI)
No recent self-harm history				
Group 1 - Liaison psychiatry service	529 (7.2)	532 (7.5)	1.04 (0.93–1.17)	1.04 (0.93–1.17)
Group 2 - Liaison nurse	81 (5.2)	117 (7.8)	1.50 (1.14–1.97)^**b**^	1.48 (1.13–1.94)^**c**^
Group 3 - No service	151 (7.9)	135 (7.0)	0.89 (0.71–1.11)	0.88 (0.71–1.10)
Recent self-harm history				
Group 1 - Liaison psychiatry service	1,714 (32.5)	1,328 (35.8)	1.10 (0.99–1.23)	1.09 (0.98–1.21)
Group 2 - Liaison nurse	209 (31.5)	376 (31.6)	1.01 (0.76–1.36)	0.99 (0.71–1.46)
Group 3 - No service	364 (35.1)	281 (30.4)	0.87 (0.66–1.13)	0.85 (0.67–1.09)

Pre-NCPSHI period was January 2012 – December 2014. Post-NCPSHI period was January 2015 – December 2017. IRR, incidence rate ratio; CI, confidence interval. *p*-values less than 0.05 are reported

a. Models adjusted for sex, age, self-harm method, alcohol involvement, brought in by ambulance, presented outside 9.00 to 17.00 h, medical card holder, medical admission, psychiatric admission, self-discharge

b. *p* = 0.004

c. *p* = 0.005

**Table 3:** Interrupted time series analysis of impact of the NCPSHI on self-harm repetition, by hospital group

	Self-harm repetition				
	Base risk IRR (95% CI)	Pre-NCPSHI trend IRR (95% CI)	Trend change IRR (95% CI)	Risk change IRR (95% CI)	Post-NCPSHI trend IRR (95% CI)
No recent self-harm history					
Group 1 - Liaison psychiatry service	0.07 (0.06–0.08)	1.00 (0.99–1.02)	0.98 (0.96–1.01)	1.14 (0.89–1.45)	0.99 (0.97-1.00)
Group 2 - Liaison nurse	0.05 (0.03–0.08)	1.00 (0.96–1.04)	1.01 (0.95–1.06)	1.35 (0.74–2.45)	1.01 (0.97–1.05)
Group 3 - No service	0.09 (0.06–0.12)	0.99 (0.96–1.02)	0.99 (0.95–1.04)	1.04 (0.65–1.68)	0.99 (0.96–1.02)
Recent self-harm history					
Group 1 - Liaison psychiatry service	0.33 (0.29–0.37)	1.00 (0.99–1.01)	1.01 (0.99–1.02)	1.04 (0.89–1.22)	1.01 (1.00-1.02)
Group 2 - Liaison nurse	0.41 (0.29–0.44)	0.97 (0.95-1.00)^a^	1.02 (0.99–1.05)	1.52 (1.02–2.27)^b^	0.99 (0.96–1.01)
Group 3 - No service	0.33 (0.27–0.41)	1.01 (0.99–1.03)	0.99 (0.96–1.02)	0.86 (0.62–1.17)	0.99 (0.97–1.02)

Pre-NCPSHI period was January 2012 – December 2014. Post-NCPSHI period was January 2015 – December 2017. Base risk refers to January-February 2012. NCPSHI was implemented in January 2015. Dependent variables were the bimonthly rates of self-harm repetition. Adjustment was made for seasonality in all models. *p*-values less than 0.05 are reported

a. *p* = 0.025

b. *p* = 0.040

### Biopsychosocial assessment

**Fig. 2:**
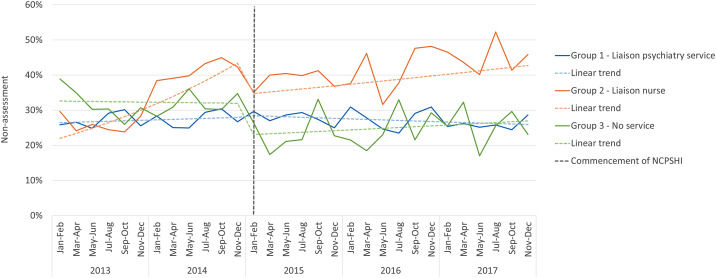
Rates of non-assessment by hospital group

Following the introduction of the NCPSHI, there was a 22% reduction in risk of non-assessment in Group 3 *No service*, at both an individual level (0.78; 0.71–0.87) and hospital level (0.72 ;0.57–0.90) (Tables 4 and 5; Fig. 2). For Group 2 *Liaison nurse*, there is evidence of an increase in risk of non-assessment following the introduction of the NCPSHI in the individual level analysis (1.21, 1.10–1.33)(Table 4). The hospital level analysis indicates that this increase reflects the attenuation of an increasing trend in non-assessment in the pre-intervention period (1.06; 1.04–1.09) (Table 5), followed by a 20% reduction (0.80; 0.92–0.98) and a trend change (0.95; 0.92–0.98) at the introduction of the NCPSHI. The pre-established increasing trend in Group 2 continued in the post NCPSHI period, but to a lesser extent (1.01; 1.00-1.03) (Table 5; Fig. 2).

**Table 4:** Poisson regression models for non-assessment in the post- versus pre-NCPSHI period, by hospital group

	Non-assessment			
	**Pre-NCPSHI** n (%)	**Post-NCPSHI** n (%)	**Unadjusted IRR** 95% CI	**Adjusted IRR** ^**a**^ 95% CI
Group 1 - Liaison psychiatry service	1,530 (27.4)	2,599 (27.1)	0.99 (0.93–1.05)	1.00 (0.94–1.06)
Group 2 - Liaison nurse	459 (33.5)	878 (41.7)	1.24 (1.13–1.37)^**b**^	1.21 (1.10–1.33)^**c**^
Group 3 - No service	507 (31.8)	701 (24.7)	0.78 (0.70–0.86)^**d**^	0.78 (0.71–0.87)^**e**^

Pre-NCPSHI period was January 2013 – December 2014. Post-NCPSHI period was January 2015 – December 2017. IRR, incidence rate ratio; CI, confidence interval. *p*-values less than 0.05 are reported. Missing data was significantly higher in the pre- compared to the post-intervention period for hospital groups 1 (20.3% vs. 11.3%) and 3 (1.5% vs. 0.4%)

a. Models adjusted for sex, self-harm method, alcohol involvement, brought by ambulance, presented outside 9.00 to 17.00 h, medical card holder, self-harm history

b-e. *p* < 0.001

**Table 5:** Interrupted time series analysis of the impact of the NCPSHI on non-assessment, by hospital group

	Non-assessment					
	Base risk (95% CI)	Pre-NCPSHI trend (95% CI)	Trend change (95% CI)	Risk change (95% CI)	Post-NCPSHI trend (95% CI)	
Group 1 - Liaison psychiatry service	0.26 (0.24–0.30)	1.00 (0.99–1.02)	0.99 (0.97–1.01)	1.02 (0.90–1.15)	0.99 (0.99-1.00)	
Group 2 - Liaison nurse	0.22 (0.18–0.27)	1.06 (1.04–1.09)^**a**^	0.95 (0.92–0.98)^**b**^	0.80 (0.92–0.98)^**c**^	1.01 (1.00-1.03)	
Group 3 - No service	0.33 (0.27–0.40)	1.00 (0.97–1.02)	1.01 (0.98–1.04)	0.72 (0.57–0.90)^**d**^	1.01 (0.99–1.02)	

Pre-NCPSHI period was January 2013 – December 2014. Post-NCPSHI period was January 2015 – December 2017. Base risk refers to January-February 2013. NCPSHI was implemented in January 2015. Dependent variables were the bimonthly rates of non-assessment. *p*-values less than 0.05 are reported

a. *p* < 0.001

b. *p* = 0.095

c. *p* = 0.042

c. *p* = 0.004

### Admission, mental health referral and self-discharge

The introduction of the NCPSHI was associated with an increase in mental health referrals (1.39; 1.26–1.53) for Group 3 *No service*, at individual level (Table 6). A decrease in medical admission was also observed for this group (0.91; 0.83-1.00). In hospital Group 2 *Liaison nurse*, there was a decrease in medical admission (0.92; 0.85–0.99) and an indication of an increase in mental health referrals (1.15; 0.98–1.35). In Group 1 *Liaison psychiatry service*, the NCPSHI was associated with a decrease in self-discharge (0.85; 0.79–0.92). Findings of hospital level analyses for these outcomes are presented in Supplementary Table 5.

**Table 6:** Poisson regression models for care pathways in the post- versus pre-NCPSHI period, by hospital group

	Pre-NCPSHI n (%)	Post-NCPSHI n (%)	Unadjusted IRR 95% CI	Adjusted IRR^a^ 95% CI
**Medical admission**				
Group 1 - Liaison psychiatry service	2,040 (18.6)	2,197 (20.4)	1.09 (1.03–1.16)^**c**^	1.05 (0.99–1.12)
Group 2 - Liaison nurse	893 (40.0)	783 (35.9)	0.90 (0.83–0.98)^**d**^	0.92 (0.83–0.97)^**e**^
Group 3 - No service	810 (27.5)	692 (24.3)	0.88 (0.80–0.97)^**f**^	0.91 (0.83-1.00)^**g**^
**Psychiatric admission**				
Group 1 - Liaison psychiatry service	894 (8.2)	889 (8.2)	1.01 (0.92–1.11)	0.99 (0.90–1.10)
Group 2 - Liaison nurse	252 (11.3)	210 (9.6)	0.85 (0.70–1.04)	0.85 (0.69–1.03)
Group 3 - No service	525 (17.8)	501 (17.6)	0.99 (0.88–1.11)	0.92 (0.82–1.03)
**Mental health referral** ^**b**^				
Group 1 - Liaison psychiatry service	1,796 (45.1)	2,405 (45.5)	1.01 (0.96–1.06)	0.99 (0.94–1.03)
Group 2 - Liaison nurse	161 (28.7)	284 (30.7)	1.07 (0.91–1.26)	1.15 (0.98–1.35)
Group 3 - No service	340 (42.2)	787 (59.0)	1.40 (1.27–1.54)^**h**^	1.39 (1.26–1.53)^**i**^
**Self-discharge**				
Group 1 - Liaison psychiatry service	1,954 (17.8)	1,599 (14.8)	0.83 (0.77–0.90)^**j**^	0.85 (0.79–0.92)^**k**^
Group 2 - Liaison nurse	305 (13.7)	300 (13.7)	1.01 (0.86–1.18)	1.06 (0.89–1.24)
Group 3 - No service	390 (13.2)	362 (12.7)	0.96 (0.84–1.10)	1.01 (0.87–1.16)

Pre-NCPSHI period was January 2013 – December 2014. Post-NCPSHI period was January 2015 – December 2017. IRR, incidence rate ratio; CI, confidence interval. *p*-values less than 0.05 are reported

a. Medical admission models adjusted for sex, age, self-harm method, alcohol involvement, brought by ambulance, presented outside 9.00 to 17.00 h, recent self-harm history; psychiatric admission and mental health referral models adjusted for sex, age, self-harm method, alcohol involvement, brought by ambulance, presented outside 9.00 to 17.00 h, medical card holder, recent self-harm history; mental health referral models adjusted for age, sex, self-harm method, alcohol involvement, brought by ambulance, presented outside 9.00 to 17.00 h, medical card holder, recent self-harm history; self-discharge models adjusted for age, sex, self-harm method, alcohol involvement, presented outside 9.00 to 17.00 h, medical card holder, recent self-harm history

b. Analyses include discharged patients only

c. *p* = 0.004

d. *p* = 0.011

e. *p* = 0.023

f. *p* = 0.012

g. *p* = 0.043

h-k. *p* < 0.001

## Discussion

We examined the impact of a national clinical programme for the assessment and management of self-harm presentations to the emergency department. The impact of the NCPSHI on self-harm repetition and provision of care components varied across hospital groups, with improvements in several aspects of care observed in those hospitals with no service for self-harm in the emergency department. The introduction of the NCPSHI in these hospitals was associated with a reduction in the risk of not receiving a biopsychosocial assessment, increased mental health referrals following discharge from the emergency department and lower rates of medical admission. Signs of a reduction in short-term self-harm repetition were also observed for this hospital group, but statistical evidence for this decrease was weak. Rates of self-discharge reduced significantly following the introduction of the NCPSHI in hospitals with a pre-existing liaison psychiatry service.

Few previous studies have examined the effectiveness of large-scale interventions aimed at improving care for patients within the emergency department. To our knowledge, there are no such studies at national level. Therefore, this study provides novel evidence that large-scale rollout of hospital-based mental health interventions across a full health service is possible and can effect meaningful change in the care provided to patients. While there is likely to be some variation in health systems in different countries, those in need of acute care for self-harm or suicidal crisis consistently present to hospital emergency departments when in need of acute care, making this evidence applicable internationally. Some studies have examined service changes at a regional or hospital level [10–12, 22, 39]. One study evaluated the impact of increased operating hours of a liaison psychiatry service in a UK hospital, with improvements in patient care reported in the short- [11] and longer-term [12]. Consistent with our findings, expansion of the liaison service was associated with improvements in the provision of assessment and referrals to other agencies, with a reduction in self-discharge. Rates of self-harm repetition within three months did not change following the expansion of the liaison service, despite the improvements in patient care [11].

The NCPSHI aimed to standardise the approach to the provision of care for self-harm patients across emergency departments nationally [9]. A central component of the model of care is a thorough biopsychosocial assessment involving an empathic, person-centred response with clear follow-up and safety planning [9]. That the greatest improvement in rates of assessment occurred in hospitals with the fewest resources for the management of self-harm prior to the intervention indicates greater standardisation across hospital groups as a result of the programme. It also indicates that ensuring that there are staff with the specific remit of assessing self-harm in place in each hospital is imperative to providing consistent high-quality care for self-harm patients. Previous research from the perspective of those with lived experience suggests that the receipt of compassionate, collaborative assessments that include aftercare planning lead to positive outcomes [23]. These characteristics are in line with the approach to assessment outlined in the NCPSHI model of care, but it was not possible to evaluate their implementation or impact in the context of the present evaluation.

It has been well-established that the provision of care for self-harm patients varies across hospitals, despite the existence of clinical guidance, which can largely be explained by the availability of specialised mental health resources within hospitals [6–8]. Therefore, it is not surprising that the impact of the programme was not uniform across hospital groups, given the difference in pre-existing services. In hospitals with a pre-existing liaison psychiatry service, there were no observed changes in assessment and repetition measures as a result of the programme’s implementation. The hospitals in this group had well-established services for assessing and treating self-harm. These hospitals were also the only group to demonstrate a reduction in rates of self-discharge from the emergency department. Allocation of additional resources may have given these teams scope to address the challenging issue of patients who leave hospital without being seen or before their care has been completed [8, 24, 25]. Mixed outcomes following the implementation of the NCPSHI were observed for hospitals with pre-existing services delivered by nurses without the support of a multidisciplinary liaison team. While the reversal of an increasing trend in non-assessments was positive, the reduction did not continue in the post-implementation period. This might be reflective of the limited out of hours cover available in these hospitals. Studies have consistently found that those presenting outside of usual working hours are less likely to receive an assessment [24, 26], often due to staff availability [27]. Furthermore, while these hospitals have a comparable number of self-harm presentations per year to the hospitals with No service, the number of nurses appointments as part of the NCPSHI was lower, meaning that these hospitals were comparably under-resourced, which is likely to impact the delivery of services [28].

Differences in the outcomes of the NCPSHI between the three hospital groups indicate differences in the implementation of the programme which may be explained by several factors. Heterogeneity in the work infrastructure and processes of care across hospitals within a health system is common and has been described specifically in relation to liaison psychiatry services in Ireland [28, 29]. There are other factors that could be hypothesised to influence implementation of such a programme, based on determinant frameworks of health services implementation, such as the support and buy-in from a range of hospital staff who have roles in referral of patients to the programme and support/supervision of the clinical nurse specialists; physical resources such as space to conduct biopsychosocial assessments and care planning; and the extent to which implementation strategies could be completed [30]. External factors, such as area level deprivation and the geographical location of the hospitals, may also contribute to the differences in the provision of care between the hospital groups [8, 31, 32]. The hospitals in group one were predominantly located urban settings, while the other two groups comprised of hospitals located outside of urban centres. This is likely to have impacted the services available within the hospitals [8], which is evident in the more comprehensive care provision for self-harm prior to the NCPSHI in hospital group one. It may also impact the availability of services to provide mental health aftercare to individuals post-discharge [31, 32]. However, our findings do not reflect this, with similar baseline rates in mental health referral in group one and three, and a substantially higher referral rate for group three in the post-implementation period. Further exploration is warranted to establish the consistency of implementation of the NCPSHI model of care across hospital sites and to understand the factors influencing implementation. The variation in resource allocation and out of hours cover across the hospital groups, and the potential impact of these differences requires specific investigation. The research team are engaging in a follow-up study to examine the determinants of implementation across hospital groups.

Whilst the present study observed some changes in self-harm repetition following the introduction of the NCPSHI, evidence of a clear association between the programme and self-harm repetition did not emerge, consistent with some other studies [11]. Hospital-presented self-harm repetition is one of the most commonly used variables to measure the efficacy of interventions to improve care for self-harm in the emergency department [11, 33, 34]. However, whether a reduction in this outcome is a valid measure of a person’s improvement has been questioned [34, 35]. People who self-harm often conceal their injuries and do not reach out to clinical services (34,35). Furthermore, people may re-attend the emergency department after a subsequent act of self-harm due to the positive support they received previously, while those who experienced negative encounters may stay away, even when intervention is needed [35, 36]. It is also possible that the introduction of the NCPSHI may have resulted in an increase in presentations of self-harm to the emergency department, due to it being the only designated service for the treatment of self-harm nationally and challenges in accessing aftercare from community providers, such as long waiting lists and narrow referral criteria [36–38]. Therefore, repeated self-harm presentations may represent a measure of clinical encounters for self-harm in the emergency department setting, rather than a true measure of self-harm repetition [39]. Even with knowledge of all repeated self-harm episodes, the validity of self-harm repetition as marker of a person’s mental state is unclear, as a decrease in the frequency of episodes can be accompanied by an increase in severity of injuries, or can lead to substitution with other negative behaviours [35]. Investigating changes in severity of repeat acts may help to provide a more complete understanding in changes in patterns of repeated self-harm acts [35]. However, this was beyond the scope of the data available for this evaluation. Information on suicide deaths would provide a more robust reflection the impact of an intervention, but sufficient data on this outcome is often not available [34], as in the present study. In the meantime, a repeated self-harm presentation to the emergency department is indicative of ongoing distress for the presenting individual [35] and is thus, still an important indicator, but one that should be interpreted with caution. In the context of the present study, the absence of a clear reduction in self-harm repetition does not indicate a failing of the NCPSHI. The appointment of specially trained nurses, equipped with a clear model of care, resulted in an increase in the provision of biopsychosocial assessments and referral to secondary mental health care. Findings from qualitative research indicate that patients find compassionate collaborate assessments and referral to appropriate aftercare services beneficial [23, 36, 40]. Determining the appropriate outcomes to evaluate mental health interventions is a challenge that is beyond the scope of this study. Patient outcomes that capture ongoing distress and help-seeking more distinctly would enhance research in this area. We also consider outcomes reflecting processes of care to be essential. However, additional research is needed to develop a core outcome set.

### Strengths and limitations

Natural experiment designs are recommended for use in real-world settings to evaluate the impact of health service initiatives in situations where randomised control trials are not feasible [22, 23]. However, such evaluations, particularly at a national scale, are relatively uncommon in the area of suicide research [31, 41, 42]. The national coverage of the Registry, providing data on all main indicators of the NCPSHI, allowed for the large-scale examination of the impact of this complex intervention across 15 hospitals of varying size and type. However, as this is an observational study, we cannot guarantee that the changes reported were caused by the NCPSHI. Furthermore, an inherent challenge when evaluating complex interventions is determining what components of the intervention are having an impact [43]. The present study examines the NCPSHI as a whole, but it is a programme with numerous active components. Considering this, as part of this programme of work, future studies will explore the implementation of the various components across hospitals and factors influencing the fidelity of implementation. Given the complexity of the intervention and the requirement for it to integrate into the emergency department setting, it is possible that the present analyses may have missed impacts of the NCPSHI that took longer to come into effect. Examining a longer follow-up period would capture changes that may have occurred after a time lag, when the intervention was embedded in routine care, but examining this was beyond the scope of the present study.

Confounding is a fundamental problem in observational studies. Of note, it was not possible to examine the impact of the NCPSHI according to suicidal intent. Variations in the provision care for self-harm patients across hospitals within the same health systems are consistently observed [6–8]. We took steps to address this in the present study, stratifying all analyses by hospital groups, with hospitals categorised according to the service that was in place prior to the implementation of NCPSHI. Given the strong association between historical and repeated acts of self-harm [2], we also stratified the analyses that examined repetition, according to self-harm history. In addition, we conducted both individual and aggregate models to enable us to report robust associations that were consistent across multiple types of analyses.

## Conclusions

Our evaluation emphasises the need for proper resourcing in order to implement clinical guidelines on the provision of care for people presenting to the emergency department with self-harm. Specifically, our findings indicate that the appointment of dedicated nurse(s), equipped with standard operating procedures, with the specific responsibility of caring for self-harm patients in the emergency department, can lead to significant service improvements. Regular training and ongoing supervision by senior clinicians are needed to support the appointed nurse(s) in the provision of comprehensive and compassionate care. The core team implementing a clinical programme such as this require buy-in across multiple settings, including the emergency department, inpatient and community mental health teams, primary care as well as other tertiary services [8, 22]. Collaboration across these settings can facilitate a coordinated response, both in terms of immediate care in the emergency department and timely and appropriate care post-discharge. This study calls into question the appropriateness of relying on self-harm repetition as the primary patient outcome in the evaluation of service provision for hospital-presented self-harm.

### Electronic supplementary material

Below is the link to the electronic supplementary material.

**Supplementary Material 1**: Unstratified results, characteristics of self-harm presentations between 2012 and 2017, comparison of characteristics of presentations before and after the NCPSHI, and interrupted time series analysis of the impact of the NCPSHI on care pathways

## Data Availability

The dataset analysed during the current study is not publicly available due to the highly sensitive nature of the data and it containing information that could compromise the privacy of individuals captured by within it, but are available from the corresponding author on reasonable request.

## Abbreviations

CI: Confidence interval
CNS: Clinical Nurse Specialist
FTE: Full time equivalent
IDO: Intentional drug overdose
IRR: Incidence rate ratio
NCPSHI: National Clinical Programme for Self-Harm and Suicide-Related Ideation
NCP-SH: National Clinical Programme for the Assessment and Management of Patients Presenting to the Emergency Department following Self-harm
Registry: National Self-Harm Registry Ireland
STROBE: Strengthening the Reporting of Observational Studies in Epidemiology
