# Supplementary material for: Evaluation of a national clinical programme for the management of self-harm in hospital emergency departments: impact on patient outcomes and the provision of care
Source: BMC Psychiatry. 2023 Dec 7;23:917. doi: 10.1186/s12888-023-05340-4 (PMC10701986; doi:10.1186/s12888-023-05340-4)
Supplement: Supplementary file 1 — Supplementary Material 1: Unstratified results, characteristics of self-harm presentations between 2012 and 2017, comparison of characteristics of presentations before and after the NCPSHI, and interrupted time series analysis of the impact of the NCPSHI on care pathways [file 12888_2023_5340_MOESM1_ESM.docx]

**Supplementary Table 1. Poisson regression models for all outcomes in the post- versus pre- NCPSHI period.**

|  | **Pre-NCPSHI**  n (%) | **Post-NCPSHI**  n (%) | **Unadjusted IRR (**95% CI) | **Adjusted IRR^a^**  (95% CI) |
| --- | --- | --- | --- | --- |
| **Self-harm repetition** | 2,508 (15.5) | 2,606 (16.5) | 1.06 (0.97-1.16) | 1.05 (0.98-1.12) |
| **Non-assessment** | 2,496 (29.2) | 4,178 (28.8) | 0.99 (0.94-1.03) | 0.99 (0.95-1.04) |
| **Medical admission** | 3,743 (23.2) | 3,672 (23.2) | 1.00 (0.96-1.05) | 0.99 (0.95-1.03) |
| **Psychiatric admission** | 1,671 (10.4) | 1,600 (10.1) | 0.98 (0.91-1.05) | 0.94 (0.87-1.01) |
| **Mental health referral^b^** | 2,297 (42.9) | 3,882 (46.0) | 1.07 (1.03-1.12)^c^ | 1.05 (1.00-1.09)^d^ |
| **Self-discharge** | 2,649 (16.4) | 2,261 (14.3) | 0.87 (0.82-0.93)^e^ | 0.89 (0.84-0.95)^f^ |

Pre-NCPSHI period was January 2012 – December 2014 for self-harm repetition, and January 2013 – December 2014 for other outcomes. Post-NCPSHI period was January 2015 – December 2017. IRR, incidence rate ratio; CI, confidence interval.

a. General admission models adjusted for sex, age, self-harm method, alcohol involvement, brought by ambulance, presented outside 9.00 to 17.00 h, recent self-harm history; psychiatric admission and mental health referral models adjusted for sex, age, self-harm method, alcohol involvement, brought by ambulance, presented outside 9.00 to 17.00 h, medical card holder, recent self-harm history; mental health referral models adjusted for age, sex, self-harm method, alcohol involvement, brought by ambulance, presented outside 9.00 to 17.00 h, medical card holder, recent self-harm history; self-discharge models adjusted for age, sex, self-harm method, alcohol involvement, presented outside 9.00 to 17.00 h, medical card holder, recent self-harm history.

b. Analyses include discharged patients only.

c. *p*=0.001

d. *p*=0.028

e-f. *p*<0.001

**Supplementary Table 2. Interrupted time series analysis of the impact of the NCPSHI on all outcomes.**

|  | **Base risk**  (95% CI) | **Pre-NCPSHI trend**  (95% CI) | **Trend change**  (95% CI) | **Risk change**  (95% CI) | **Post-NCPSHI trend** (95% CI) |
| --- | --- | --- | --- | --- | --- |
| **Self-harm repetition** | 0.16 (0.15-0.17) | 1.00 (0.99-1.01) | 1.00 (0.99-1.01) | 1.11 (1.00-1.24) | 1.00 (0.99-1.00) |
| **Non-assessment** | 0.27 (0.24-0.29) | 1.01 (1.00-1.03) | 0.99 (0.97-1.00) | 0.92 (0.83-1.01) | 1.00 (0.99-1.00) |
| **Medical admission** | 0.22 (0.20-0.24) | 1.00 (0.99-1.01) | 1.01 (1.00-1.02) | 0.97 (0.88-1.08) | 1.01 (1.00-1.02)^b^ |
| **Psychiatric admission** | 0.10 (0.89-0.12) | 1.01 (0.99-1.02) | 0.97 (0.95-0.99)^d^ | 1.10 (0.95-1.28) | 0.98 (0.97-0.99)^e^ |
| **Mental health referral^a^** | 0.38 (0.34-0.41) | 1.01 (1.01-1.03) | 0.97 (0.96-0.99)^i^ | 1.04 (0.94-1.15) | 0.99 (0.99-1.00) |
| **Self-discharge** | 0.18 (0.17-0.20) | 0.99 (0.98-1.00) | 1.00 (0.99-1.02) | 0.97 (0.86-1.09) | 0.99 (0.98-1.00) |

Pre-NCPSHI period was January 2012 – December 2014 for self-harm repetition, and January 2013 – December 2014 for other outcomes. Post-NCPSHI period was January 2015 – December 2017. Base rate refers to January-February in the first year of the pre-NCPSHI period. NCPSHI was implemented in January 2015. Dependent variables were rates of medical admission, psychiatric admission, mental health referral and self-discharge. Mental health referral models based on individuals who were discharged from the emergency department. *p*-values less than 0.05 are reported. Adjustment was made for seasonality in self-harm repetition model.

a. Analyses include discharged patients only.

b. *p*<0.001

c. *p*<0.001

d. *p*=0.021

e-j. *p*<0.001

k. *p*=0.015

**Supplementary Table 3. Individual characteristics of self-harm presentations between 2012 and 2017.**

| Variables | All | | |
| --- | --- | --- | --- |
|  | n | % | |
| All | 31,970 | 100 | |
| Sex |  |  | |
| Male | 15,285 | 47.8 | |
| Female | 16,685 | 52.2 | |
| Median age in years (IQR) | 33 | 21 | |
| Method of self-harm |  |  | |
| Intentional drug overdose (IDO) only | 19,146 | 59.9 | |
| Self-cutting only | 5,833 | 18.3 | |
| IDO and self-cutting | 1,282 | 4.0 | |
| Attempted hanging | 1,557 | 4.9 | |
| Attempted drowning | 843 | 2.6 | |
| Other methods | 3,309 | 10.4 | |
| Alcohol involvement | 11,666 | 36.5 | |
| Brought in by ambulance | 17,724 | 57.7 | |
| Presented outside 9.00 to 17.00 hours | 9,453 | 29.6 | |
| Recent self-harm history | 10,631 | 33.3 | |
| Medical card holder^1^ | 13,611 | 42.6 | |
| Residence status |  |  | |
| Household resident | 30,082 | 94.1 | |
| Hospital in-patient | 127 | 0.4 | |
| Prisoner | 152 | 0.5 | |
| No fixed abode recorded | 1,017 | 3.2 | |
| Other | 533 | 1.7 | |
| 3-month self-harm repetition | 5,114 | 16.0 | |
| Repetition with no recent self-harm history | 1,545 | 7.2 | |
| Repetition with recent self-harm history | 3,569 | 33.5 | |
| Non-assessment^2^ | 6,674 | 28.9 | |
| Medical admission | 7,415 | 23.2 | |
| Psychiatric admission | 3,271 | 10.2 | |
| Mental health referral^3^ | 6,179 | 44.8 | |
| Self-discharge | 4,910 | 15.4 | |
| ^1^Medical card holder unknown for 32.7% of presentations.  ^2^Assessment unknown for 10.8% of presentations.  ^3^Percentage based on those discharged home from the emergency department. | | |  |

**Supplementary Table 4. Comparison of characteristics of self-harm presentations before (2012-2014) and after the NCPSHI (2015-2017).**

| Variables | **Group 1 - Liaison psychiatry service** | | | | | **Group 2- Liaison nurse** | | | | | **Group 3 - No service** | | | | |
| --- | --- | --- | --- | --- | --- | --- | --- | --- | --- | --- | --- | --- | --- | --- | --- |
|  | **Pre-NCPSHI (2012-2014)** | | **Post-NCPSHI (2015-2017)** | | ***p*** | **Pre-NCPSHI (2012-2014)** | | **Post-NCPSHI (2015-2017)** | | ***p*** | **Pre-NCPSHI (2012-2014)** | | **Post-NCPSHI (2015-2017)** | | ***p*** |
|  | **n** | **%** | **n** | **%** |  |  | **%** | **n** |  |  | **n** | **%** | **n** | **%** |  |
| Number of presentations | 10,956 | 50.4 | 10,798 | 49.6 |  | 2,235 | 50.6 | 2,183 | 49.4 |  | 2,949 | 50.9 | 2,849 | 49.1 |  |
| Sex |  |  |  |  |  |  |  |  |  |  |  |  |  |  |  |
| Male | 5,239 | 47.8 | 5,103 | 47.3 |  | 1,048 | 46.9 | 990 | 45.4 |  | 1,481 | 50.2 | 1,424 | 50.0 |  |
| Female | 5,717 | 52.2 | 5,695 | 52.7 | 0.408 | 1,187 | 53.1 | 1,193 | 54.7 | 0.305 | 1,468 | 49.8 | 1,425 | 50.0 | 0.856 |
| Age group |  |  |  |  |  |  |  |  |  |  |  |  |  |  |  |
| <15 years |  |  |  |  |  |  |  |  |  |  |  |  |  |  |  |
| 15-24 years | 2,830 | 25.8 | 2,914 | 27.0 |  | 502 | 22.5 | 503 | 23.0 |  | 755 | 25.6 | 739 | 25.9 |  |
| 25-44 years | 5,533 | 50.5 | 5,260 | 48.7 |  | 1,056 | 47.3 | 995 | 45.6 |  | 1,413 | 47.9 | 1,404 | 49.3 |  |
| 45-64 years | 2,286 | 20.9 | 2,274 | 21.1 |  | 604 | 27.0 | 615 | 28.2 |  | 718 | 24.4 | 637 | 22.4 |  |
| 65+ years | 307 | 2.8 | 350 | 3.2 | 0.020 | 73 | 3.3 | 70 | 3.2 | 0.714 | 63 | 2.1 | 69 | 2.4 | 0.309 |
| Method of self-harm |  |  |  |  |  |  |  |  |  |  |  |  |  |  |  |
| Intentional drug overdose (IDO) only | 6,549 | 59.8 | 6,192 | 57.3 |  | 1,514 | 67.7 | 1,431 | 65.6 |  | 1,800 | 61.0 | 1,660 | 58.3 |  |
| Self-cutting only | 2,026 | 18.5 | 2,211 | 20.5 |  | 317 | 14.2 | 335 | 15.4 |  | 453 | 15.4 | 491 | 17.2 |  |
| IDO and self-cutting | 463 | 4.2 | 466 | 4.3 |  | 74 | 3.3 | 95 | 4.4 |  | 99 | 3.4 | 85 | 3.0 |  |
| Attempted hanging | 473 | 4.3 | 511 | 4.7 |  | 97 | 4.3 | 104 | 4.8 |  | 191 | 6.5 | 181 | 6.4 |  |
| Attempted drowning | 324 | 3.0 | 324 | 3.0 |  | 37 | 1.7 | 36 | 1.7 |  | 58 | 2.0 | 64 | 2.3 |  |
| Other methods | 1,121 | 10.2 | 1,094 | 10.1 | 0.002 | 196 | 8.8 | 182 | 8.3 | 0.346 | 348 | 11.8 | 368 | 12.9 | 0.176 |
| Alcohol involvement | 3,839 | 35.0 | 3,290 | 30.5 | <0.001 | 1,201 | 53.7 | 1,057 | 48.4 | <0.001 | 1,271 | 43.1 | 1,008 | 35.4 | <0.001 |
| Brought in by ambulance | 6,165 | 63.1 | 5,315 | 49.5 | <0.001 | 1,294 | 58.3 | 1,300 | 59.6 | 0.385 | 1,923 | 65.4 | 1,727 | 60.6 | <0.001 |
| Presented outside 9.00-17.00 hours | 3,074 | 28.1 | 3,449 | 32.0 | <0.001 | 647 | 29.0 | 606 | 27.8 | 0.381 | 824 | 28.0 | 853 | 30.0 | 0.095 |
| Recent self-harm history | 3,615 | 33.0 | 3,709 | 34.4 | 0.035 | 671 | 30.0 | 674 | 30.9 | 0.538 | 1,038 | 35.2 | 924 | 32.4 | 0.026 |
| Medical card holder | 2,757 | 25.2 | 2,755 | 25.5 | 0.065 | 1,060 | 47.4 | 1,056 | 48.4 | 0.012 | 1,633 | 55.4 | 1,631 | 57.3 | <0.001 |
| Residence status |  |  |  |  |  |  |  |  |  |  |  |  |  |  |  |
| Household resident | 10,287 | 93.9 | 9,907 | 91.8 |  | 2,154 | 96.4 | 2,106 | 96.5 |  | 2,854 | 96.8 | 2,774 | 97.4 |  |
| Hospital in-patient | 30 | 0.3 | 49 | 0.5 |  | 17 | 0.8 | 6 | 0.3 |  | 17 | 0.6 | 8 | 0.3 |  |
| Prisoner | 52 | 0.5 | 42 | 0.4 |  | 32 | 1.4 | 20 | 0.9 |  | 2 | 0.1 | 4 | 0.1 |  |
| No fixed abode recorded | 374 | 3.4 | 560 | 5.2 | <0.001 | 18 | 0.8 | 22 | 1.0 | 0.013 | 29 | 1.0 | 14 | 0.5 | 0.099 |

**Supplementary Table 5. Interrupted time series analysis of the impact of the NCPSHI on care pathways, by hospital group.**

|  | **Base risk**  (95% CI) | **Pre-NCPSHI trend**  (95% CI) | **Trend change**  (95% CI) | **Risk change**  (95% CI) | **Post-NCPSHI trend** (95% CI) |
| --- | --- | --- | --- | --- | --- |
| **Medical admission** |  |  |  |  |  |
| Group 1 - Liaison psychiatry service | 0.17 (0.15-0.20) | 1.00 (0.98-1.02) | 1.02 (1.00-1.04) | 1.04 (0.90-1.19) | 1.02 (1.01-1.02)^b^ |
| Group 2 - Liaison nurse | 0.40 (0.33-0.47) | 0.99 (0.97-1.02) | 1.01 (0.73-1.13) | 0.90 (0.73-1.13) | 1.01 (0.99-1.02) |
| Group 3 - No service | 0.23 (0.19-0.28) | 1.02 (0.99-1.04) | 0.98 (0.95-1.01) | 0.86 (0.69-1.08) | 1.00 (0.99-1.01) |
| **Psychiatric admission** |  |  |  |  |  |
| Group 1 - Liaison psychiatry service | 0.07 (0.06-0.08) | 1.03 (1.00-1.05)^c^ | 0.94 (0.91-0.96)^d^ | 1.19 (0.98-1.44) | 0.96 (0.95-0.98)^e^ |
| Group 2 - Liaison nurse | 0.12 (0.09-0.17) | 0.98 (0.94-1.03) | 1.02 (0.96-1.07) | 0.97 (0.64-1.48) | 1.00 (0.97-1.03) |
| Group 3 - No service | 0.21 (0.17-0.26) | 0.99 (0.96-1.02) | 1.02 (0.98-1.05) | 0.98 (0.75-1.28) | 1.00 (0.99-1.02) |
| **Mental health referral ^b^** |  |  |  |  |  |
| Group 1 - Liaison psychiatry service | 0.40 (0.36-0.44) | 1.02 (1.00-1.03)^f^ | 0.97 (0.96-0.99)^g^ | 1.01 (0.90-1.14) | 0.99 (0.98-1.00)^h^ |
| Group 2 - Liaison nurse | 0.32 (0.23-0.44) | 0.98 (0.94-1.03) | 1.01 (0.96-1.06) | 1.26 (0.86-1.85) | 0.99 (0.96-1.02) |
| Group 3 - No service | 0.31 (0.24-0.40) | 1.05 (1.01-1.08)^i^ | 0.96 (0.93-0.99)^j^ | 1.07 (0.84-1.37) | 1.00 (0.99-1.02) |
| **Self-discharge** |  |  |  |  |  |
| Group 1 - Liaison psychiatry service | 0.20 (0.18-0.22) | 0.99 (0.97-1.00) | 1.00 (0.99-1.02) | 0.94 (0.81-1.08) | 0.99 (0.98-1.00) |
| Group 2 - Liaison nurse | 0.13 (0.09-0.17) | 1.01 (0.97-1.05) | 0.97 (0.93-1.02) | 01.25 (0.88-1.78) | 0.98 (0.96-1.00) |
| Group 3 - No service | 0.17 (0.13-0.22) | 0.98 (0.95-1.01) | 1.01 (0.98-1.06) | 0.96 (0.70-1.31) | 1.00 (0.98-1.02) |

Pre-NCPSHI period was January 2013 – December 2014. Post-NCPSHI period was January 2015 – December 2017. Dependent variables were rates of medical admission, psychiatric admission, mental health referral and self-discharge. Mental health referral models based on individuals who were discharged from the emergency department. *p*-values less than 0.05 are reported.

a. Analyses include discharged patients only.

b. *p*<0.001

c. *p*=0.019

d-e. *p*<0.001

f. *p*=0.011

g. *p*<0.001

h. *p*=0.005

i. *p*=0.005

j. *p*=0.017

k. *p*=0.040
